# Changes in Spatial Patterns of *Caragana stenophylla* along a Climatic Drought Gradient on the Inner Mongolian Plateau

**DOI:** 10.1371/journal.pone.0121234

**Published:** 2015-03-18

**Authors:** Li-Na Xie, Hong-Yu Guo, Christopher A. Gabler, Qing-Fang Li, Cheng-Cang Ma

**Affiliations:** 1 Tianjin Key Laboratory of Animal and Plant Resistance, College of Life Sciences, Tianjin Normal University, Tianjin, P.R. China; 2 Department of Biology and Biochemistry, University of Houston, Houston, Texas, United States of America 77204; University of New South Wales, AUSTRALIA

## Abstract

Few studies have investigated the influence of water availability on plant population spatial patterns. We studied changes in the spatial patterns of *Caragana stenophylla* along a climatic drought gradient within the Inner Mongolian Plateau, China. We examined spatial patterns, seed density, “nurse effects” of shrubs on seedlings, transpiration rates and water use efficiency (WUE) of *C*. *stenophylla* across semi-arid, arid, and intensively arid zones. Our results showed that patches of *C*. *stenophylla* populations shifted from a random to a clumped spatial pattern towards drier environments. Seed density and seedling survival rate of *C*. *stenophylla* decreased from the semi-arid zone to the intensively arid zone. Across the three zones, there were more *C*. *stenophylla* seeds and seedlings underneath shrub canopies than outside shrub canopies; and in the intensively arid zone, there were almost no seeds or seedlings outside shrub canopies. Transpiration rates of outer-canopy leaves and WUE of both outer-canopy and inner-canopy leaves increased from the semi-arid zone to the intensively arid zone. In the intensively arid zone, transpiration rates and WUE of inner-canopy leaves were significantly lower and higher, respectively, than those of outer-canopy leaves. We conclude that, as drought stress increased, seed density decreased, seed proportions inside shrubs increased, and “nurse effects” of shrubs on seedlings became more important. These factors, combined with water-saving characteristics associated with clumped spatial patterns, are likely driving the changes in *C*. *stenophylla* spatial patterns.

## Introduction

Population spatial patterns of plants can vary with environmental conditions. Studies have shown that facilitation from nurse plants in stressful environments [1], seasonality, drought [2], human disturbance [3], presence or absence of competitors [4, 5], frugivore diversity and behavior [6], and life history stages [7] could be related to changes in the type of spatial patterns (i.e., random, regular or clumped) exhibited by plant populations. Climatic drought stress is one of the important factors affecting plant growth, development and distribution, but few studies have investigated the influence of a climatic drought gradient on plant population spatial patterns along that gradient. Sun et al (1994)[8] studied the woody plant *Betula pianyphylla*, and found that its population spatial patterns changed from concentrated to random type as humidity decreased or temperature increased. Malkinson & Jeltsch (2007) [9] found that the shrub *Sarcopoterium spinosum* exhibited clumped spatial patterns in moist fields, but had random spatial patterns in arid sites where precipitation was lower. Li et al. (2009) [10] reported that spatial patterns of the sub-shrub *Artemisia ordosica* changed from random to clumped types as precipitation decreased. However, development of a more general and mechanistic understanding of how climate, especially drought, influences plant population spatial patterns requires studies on more species in different ecosystems.

The climate on the Inner Mongolian Plateau is characterized by a gradual increase in solar radiation and air temperature, and a gradual decrease in precipitation, from the northeast to the southwest. Decreased precipitation together with increased evaporation results in a strong climatic drought gradient from the northeast to the southwest. Seven moisture zones are defined within the Inner Mongolian Plateau and include, in order from northeast to southwest: humid, sub-humid, semi-arid, arid, very arid, intensively arid and extremely arid zones [11]. Vegetation coverage decreases gradually and desertification becomes more severe along the same northeast to southwest gradient. Seven vegetation types are associated with these seven moisture zones: forest, meadow steppe, typical steppe, desertification steppe, steppe desert, typical desert and extremely dry desert. With its strong climatic drought gradient, the Inner Mongolian Plateau provides an ideal system for studying plant adaptations to drought stress.

*Caragana* species are deciduous shrubs widely distributed in grassland and desert ecosystems in semiarid and arid areas in Asia and Europe. There are more than 100 species in the genus of *Caragana*, and there are 62 *Caragana* species in China. *Caragana* species are well known for their drought resistance; they can survive under severe drought conditions and are called “life-saving plants” for livestock [12]. There are 16 *Caragana* species distributed on the Inner Mongolian Plateau, and *Caragana stenophylla* is one of the *Caragana* species with important ecological functions in the region [13, 14]. *C*. *stenophylla* is distributed across a large geographic range on the Inner Mongolian Plateau, predominately from the semi-arid zone to the intensively arid zone.

We previously reported the geographic distribution of *C*. *stenophylla* and its morphological, physio-ecological and reproductive adaptations to drought in the region [14–19], but little is known about the spatial patterns of *C*. *stenophylla* along the drought gradient of the Inner Mongolian Plateau, or the factors driving these patterns. We hypothesized that drought stress promotes clumped spatial patterns within *C*. *stenophylla* populations over random spatial patterns, which are favored in wetter environments. Further, we hypothesized that changes in recruitment strategies and adaptations to local environments might be the main factors driving changes in the spatial patterns of *C*. *stenophylla*.

To test our hypotheses, we investigated *C*. *stenophylla* populations across the climatic drought gradient represented by the semi-arid, arid and intensively arid zones on the Inner Mongolian Plateau. Specifically, we examined and compared the spatial patterns, seed densities, “nurse effects” of shrubs on seedlings, transpiration rates and water use efficiency (WUE) of *C*. *stenophylla* populations within these focal climate zones.

## Methods

### Study sites

We conducted the field study in Xilinhaote (with permission from Xilinhaote City Grassland Work Station) in the semi-arid zone, Suniteyou (with permission from Suniteyou Banner [county equivalent] Grassland Work Station) in the arid zone, and Alashanzuo (with permission from Alashanzuo Banner Grassland Work Station) in the intensively arid zone of the Inner Mongolia Plateau. Environmental data for the study sites are shown in Table 1. Field surveys and experiments were conducted in 2012, 2013 and 2014. Within each site, we identified three study plots that were 20–40 km apart for field surveys and experiments (Table 1). *C*. *stenophylla* is not an endangered or threatened species at the study sites.

**Table 1:** Environmental data of the study sites.

Site	Plot longitude and latitude	Altitude (m)	Annual Average precipitation (mm)	Annual average temperature (°C)	Sunshine duration (h)	Moisture type	Vegetation type	Vegetation coverage (%)
Xilinhaote	N43°55′20" E115°32′42", N44°28′31" E115°55′19", N44°15′19" E115°53′39"	990	281	2.35	2932	Semi-arid	Steppe	25–50%
Suniteyou	N42°20′59" E112°56′43", N42°27′58" E112°48′40", N42°47′18" E112°32′10"	1151	211	4.93	3167	Arid	Desertification steppe	10–35%
Alashanzuo	N38°23′39" E105°38′11", N38°19′47" E105°41′34", N38°30′38" E105°42′12"	1561	110	7.80	3200	Intensively arid	Desert	1–20%

Climate data are the mean values over 40 years.

### Table Field surveys and experiments

#### Point pattern analysis

At each site, we randomly selected one of the three plots for point pattern analysis. Within these plots, we set up a 40 m×40 m quadrat and divided it into 1600 sub-quadrats (1m×1m). We then quantified and further investigated the *C*. *stenophylla* individuals in each sub-quadrat. First, for each *C*. *stenophylla* shrub (or shrub cluster) in a sub-quadrat, we carefully removed the sand dune and excavated 20–30 cm below the soil surface in order to expose the belowground structures of the shrub (or shrub cluster) and identify all individuals (Arising from either sexual reproduction or asexual reproduction (ramets)). Second, we recorded the coordinate position of each *C*. *stenophylla* individual and analyzed the position data for each 40 m×40 m quadrat using point pattern analyses, based on the linearized form of Ripley's *K*-function. Point pattern analyses were performed using the software Programita (2008) [20, 21]. Spatial scale was 0–20 m, and scale point size was 0.5 m. For each 40 m×40 m quadrat, we calculated approximate 99% confidence envelopes (confidence intervals) using a Monte-Carlo simulation test with 99 randomizations. If the spatial pattern of shrubs within a quadrat is random, calculated *K*(*t*) values would be within the confidence envelope; however, if the spatial pattern is clumped or uniform, *K*(*t*) values would be either above the upper bound or below the lower bound of the envelope, respectively [22].

To complement our point-pattern analysis, we sampled ten *C*. *stenophylla* shrubs (or shrub clusters) in each plot at each site (n = 30 shrubs or shrub clusters per site) to quantify the aggregation extent using the line transect method. For each shrub (or shrub cluster), we removed the sand dune and excavated 20–30 cm of belowground structures of the shrub (or shrub cluster) to determine whether it was a single individual (accompanied by no other individuals from either sexual or asexual reproduction), a homologous cluster (consisted of asexually reproduced individuals (ramets) of the same clone, ramets were connected by horizontal roots), or a heterologous cluster (consisted of individuals either sexually or asexually reproduced of different clones). For each study site, we recorded the number of single individuals, homologous clusters and heterologous clusters, and calculated the proportion of each type. For each plot within the sites, we also recorded the number of individuals from sexual reproduction, the number of individuals from asexual reproduction (ramets), and the total number of individuals within each shrub cluster, and we calculated the average values of these variables for each plot. We used the number of individuals per cluster and the proportions of single individuals, homologous clusters and heterologous clusters to quantify population aggregation extent at each study site.

#### Seed distribution

In each study plot, we randomly sampled five *C*. *stenophylla* shrubs (or shrub clusters) and quantified the distributions of seeds inside and outside the shrub canopies (n = 15 shrubs or shrub clusters per site). For each shrub (or shrub cluster), we collected soil samples (50 cm long × 50 cm wide × 5 cm deep) both below the shrub canopy (inside-shrub) and 2 m away from the edge of the shrub canopy (outside-shrub). We collected *C*. *stenophylla* seeds by sieving (mesh size 1.25 mm) soil samples, and we recorded the number of *C*. *stenophylla* seeds and calculated seed density for each soil sample.

#### Survival rate of seedlings

During June-July, 2012, we collected healthy *C*. *stenophylla* seeds from plants in a single location at each of the three study sites respectively, allowed these seeds to germinate and grow in sand media for 40 days in a light incubator (12 hr light/12 hr dark; 25°C/15°C). At the beginning of the 2013 growing season (*Caragana* budding), we transplanted the seedlings back into the plots at the sites where the seeds were collected. The timing of the growing season varies significantly among our study sites, so we transplanted the seedlings accordingly on different dates for different sites. The transplant dates were May 1 at Alashanzuo, May 15 at Suniteyou, and May 25 at Xilinhaote. At ten shrubs (or shrub clusters) in each study plot, we transplanted 20 seedlings inside and 20 seedlings outside each shrub canopy (n = 400 seedlings per plot, 1200 seedlings per site). In September 2013 (the end of the growing season, i.e. when *Caragana* leaves turned yellow), we quantified seedling survival both inside and outside the shrub canopies for each plot at each study site.

#### Transpiration rate and water use efficiency

In August 2013, we sampled ten shrubs (or shrub clusters) in each plot (n = 30 shrubs or shrub clusters per site), using the line transect method, to quantify net photosynthetic rate and transpiration rate with a LI-6400 Portable Photosynthesis System (LI-COR Co., USA). For each shrub, we conducted two measurements (one on inner-canopy leaves and one on outer-canopy leaves) every 2 hours from 7:00–19:00 during a sunny day (3 sunny days at each study site). We used these measurements to calculate daily average net photosynthesis and transpiration rates for inner- and outer-canopy leaves for each plot. Daily transpiration values were calculated using the formula: Daily transpiration value = daily average transpiration rate (mol H_2_O·m^-2^·s^-1^) × 3600 (s/hr) × 12 (hr). Water use efficiency (WUE) was calculated according to the formula: WUE = daily average net photosynthesis rate / daily average transpiration rate.

### Data analysis

Data analyses were performed with SPSS 16.0 (SPSS Inc). We used Chi-square (*χ* ^2^) tests to examine the differences in the numbers of single individuals, homologous clusters and heterologous clusters among the three different zones. We performed one-way ANOVAs and Tukey HSD tests to examine the differences in the abundance of individuals from sexual reproduction, abundance of individuals from asexual reproduction, seed density, seedling survival rate, transpiration rate and WUE among the three different zones (n = 3 (averages for each plot) in each zone). Within each zone, we used t-tests to compare the means of seed density and seedling survival rates inside versus outside shrubs, and to compare the means of transpiration rates and WUE for inner-canopy versus outer-canopy leaves.

## Results

### Spatial patterns

The spatial patterns of *C*. *stenophylla* differed among the three zones. In the semi-arid zone, *C*. *stenophylla* exhibited random spatial patterns at all scales except for the 1 m scale, where it was clumped (S1a Fig.). In the arid zone, *C*. *stenophylla* exhibited clumped spatial patterns at the 1–2 m and 5–9 m scales (S1b Fig.). In the intensively arid zone, *C*. *stenophylla* exhibited clumped spatial patterns in the 1–12 m scale ranges (S1c Fig.).

**Fig 1:**
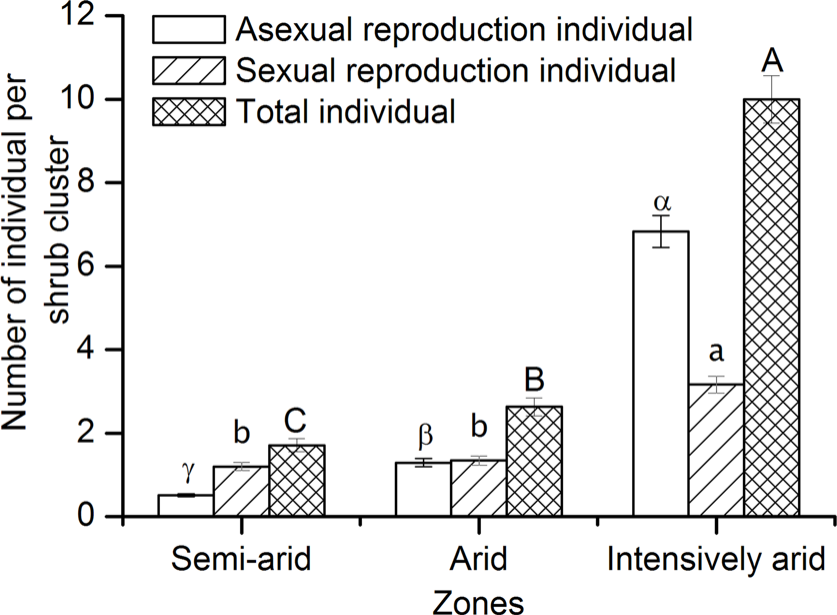
Average abundances of individuals arising from sexual or asexual reproduction and average total numbers of individuals for *C*. *stenophylla* shrub clusters in the semi-arid, arid and intensively arid zones (mean ± SE, n = 3). For variables in the same category, different letters (asexual reproduction individual: greek letter; sexual reproduction individual: lowercase; total number of individual: uppercase) indicate significant differences in the means between zones (Tukey HSD tests, *P* < 0.05).

In the semi-arid zone, 76.7% of *C*. *stenophylla* plants grew as single individuals, 10.0% formed homologous clusters, and 13.3% formed heterologous clusters. In the arid zone, the proportions of single individuals, homologous clusters and heterologous clusters were 50.0%, 33.3% and 16.7%, respectively. In the intensively arid zone, these proportions were 0.0%, 40.0% and 60.0%, respectively. From the semi-arid zone to the intensively arid zone, the proportions of shrubs present individually decreased and the proportions of shrubs present in either cluster type increased (Table 2). The total number of individuals per shrub cluster and abundances of individuals arising both sexually and asexually per cluster increased from the semi-arid zone to the intensively arid zone (Fig. 1). These results demonstrate that aggregation of *C*. *stenophylla* populations increased from the semi-arid zone to the intensively arid zone.

**Table 2:** Abundances and proportions (in parentheses) of single individuals, homologous clusters and heterologous clusters of *C*. *stenophylla* in the semi-arid, arid and intensively arid zones.

Shrub composition	Semi-arid zone	Arid zone	Intensively arid zone
Single individual	23 (76.7%)	15(50.0%)	0 (0%)
Homologous clusters	3 (10.0%)	10 (33.3%)	12 (40%)
Heterologous clusters	4 (13.3%)	5 (16.7%)	18 (60%)
*χ* ^2^ test results	*χ* ^2^_4_ = 40.442 *P*<0.01		

### Seed distribution

**Fig 2:**
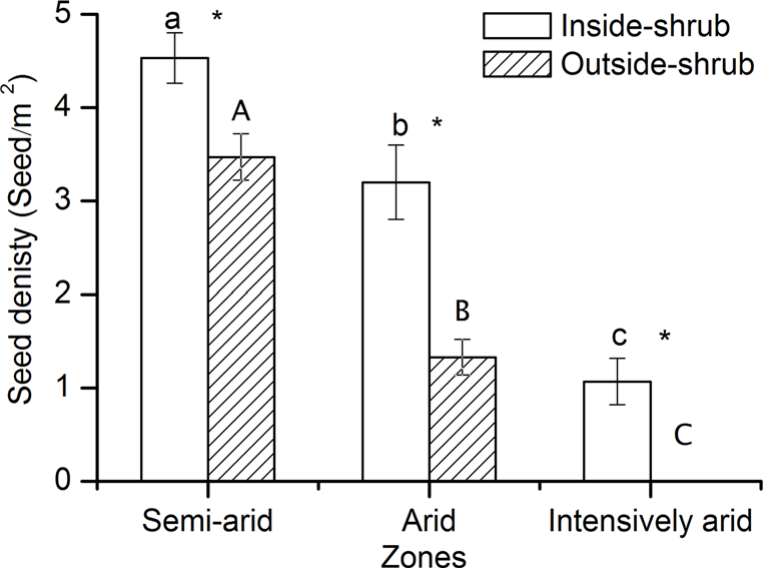
Seed densities inside and outside shrub canopies of *C*. *stenophylla* in the semi-arid, arid and intensively arid zones (mean ± SE, n = 3). For variables in the same category, different letters (inside-shrub: lowercase; outside-shrub: uppercase) indicate significant differences in the means between zones (Tukey HSD tests, *P* < 0.05). Within each zone, asterisks indicate significant differences in the means between inside-shrub and outside-shrub (t-test, *P* < 0.05).

Seed density of *C*. *stenophylla* decreased from the semi-arid to the intensively arid zone. Inside shrub canopies, the seed density in the semi-arid zone was 1.41 and 4.23 times higher than in the arid zone and intensively arid zone, respectively. Outside shrub canopies, the seed density in the semi-arid zone was 2.61 time higher than in the arid zone. For all three climate zones, seed density was higher inside shrub canopies than outside shrub canopies. In the semi-arid and arid zones, seed densities were 1.31 and 2.41 times higher inside shrub canopies than outside shrub canopies, respectively. In the intensively arid zone, seeds were only observed inside shrub canopies (Fig. 2).

### Nurse effects of shrubs on seedlings

**Fig 3:**
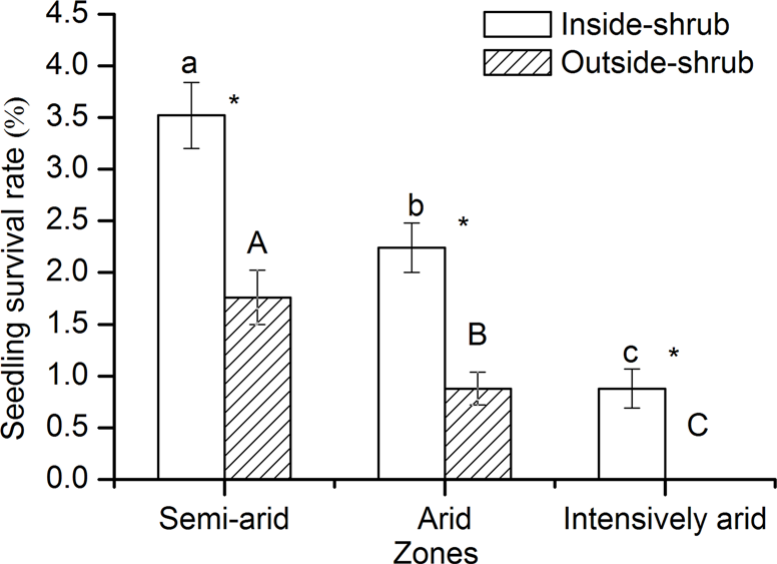
Seedling survival rates inside and outside shrub canopies of *C*. *stenophylla* in the semi-arid, arid and intensively arid zones (mean ± SE, n = 3). For variables in the same category, different letters (inside-shrub: lowercase; outside-shrub: uppercase) indicate significant differences in the means between zones (Tukey HSD tests, *P* < 0.05). Within each zone, asterisks indicate significant differences in the means between inside-shrub and outside-shrub (t-test, *P* < 0.05).

Survival rates of *C*. *stenophylla* seedlings decreased from the semi-arid zone to the intensively arid zone. In all three climate zones, seedling survival rates were higher inside shrub canopies than outside shrub canopies (*P* < 0.05). In the semi-arid and arid zones, seedling survival rates were 2.00 and 2.55 times higher inside shrub canopies versus outside shrub canopies, respectively (*P* < 0.05). In the intensively arid zone, no seedlings survived outside shrub canopies (Fig. 3).

### Water consumption

The daily transpiration of inner-canopy leaves did not significantly differ among the focal climate zones. However, the daily transpiration of outer-canopy leaves increased from the semi-arid zone to the intensively arid zone. In the semi-arid zone, the daily transpiration did not differ between inner- and outer-canopy leaves. However, the daily transpiration of inner-canopy leaves tended to be lower than that of outer-canopy leaves in the arid zone, and was significantly lower than that of outer-canopy leaves in the intensively arid zone (Table 3).

**Table 3:** Transpiration rates and water use efficiency (WUE) of inner-canopy and outer-canopy leaves of *C*. *stenophylla* in the semi-arid, arid and intensively arid zones (n = 3).

		Semi-arid zone	Arid zone	Intensively arid zone
Daily transpiration	Outer-canopy leaves (molH_2_O·m^-2^·d^-1^)	244.2b	251.4ab	265.0a
	Inner-canopy leaves (molH_2_O·m^-2^·d^-1^)	243.8A	245.6A	248.7A*
	Differences between inner-canopy and outer-canopy leaves (%)	0.2	2.3	6.2
WUE	Outer-canopy leaves (mmolCO_2_· mol^-1^ H_2_O)	1.48b	1.51b	1.71a
	Inner-canopy leaves (mmolCO_2_· mol^-1^ H_2_O)	1.48B	1.55B	1.81A*
	Differences between inner-canopy and outer-canopy leaves (%)	0.3	2.6	5.8

For the same variable, different letters (outer-canopy leaves: lowercase; inner-canopy leaves: uppercase) indicate significant differences in the means between zones (Tukey HSD tests, *P* < 0.05). Within each zone, asterisks indicate significant differences in the means between inner-canopy leaves and outer-canopy leaves (t-test, *P* < 0.05).

Water use efficiency (WUE) of *C*. *stenophylla* increased from the semi-arid zone to the intensively arid zone. In the semi-arid zone, WUE did not significantly differ between inner-canopy leaves and outer-canopy leaves. In the arid zone, WUE tended to be higher among inner-canopy leaves than for outer-canopy leaves. In the intensively arid zone, WUE of inner-canopy leaves was significantly higher than that of outer-canopy leaves (Table 3).

## Discussion

Climate is one of the primary factors governing plant spatial patterns. Results of point pattern analyses showed that *C*. *stenophylla* individuals were distributed randomly in the semi-arid zone, but had clumped spatial patterns at some scales in the arid zone, and at an even broader range of scales in the intensively arid zone. We also found that aggregation within *C*. *stenophylla* populations increased from the semi-arid zone to the intensively arid zone. These findings suggest that the spatial patterns of *C*. *stenophylla* transition from random to clumped types as climatic drought stress increases. Similar changes in spatial patterns were documented in *Artemisia ordosica* populations [10]. In the semi-arid zone, random spatial patterns within *C*. *stenophylla* populations were associated with relatively small shrub (or shrub cluster) sizes (average canopy area: 0.204 m^2^), whereas, in the intensively arid zone, clumped spatial patterns were associated with considerably larger shrub cluster sizes (average canopy area: 2.979 m^2^) [14].

Several studies on other plant species have found that seed density and seedling survival beneath canopies are much higher than in open patches [23–30]. Our results showed that *C*. *stenophylla* seed density was much higher inside shrub canopies than outside shrub canopies, and the proportion of seeds outside shrub canopies decreased as drought stress increased. In the semi-arid zone, *C*. *stenophylla* seeds were common, seeds could germinate, and seedlings were able to survive both inside and outside of shrub canopies. This permitted establishment of a large number of scattered single individuals. As drought stress increased, both the number of *C*. *stenophylla* seeds and seedling survival rates decreased. In the intensively arid zone, *C*. *stenophylla* seeds were only observed inside shrub canopies and no transplanted seedlings survived outside shrub canopies. This may be because physical conditions (e.g., wind speed, humidity, temperature, soil moisture, soil fertility, etc.) were relatively favorable for seedlings inside shrub canopies in the intensively arid zone compared to outside shrub canopies [25, 31–33]. Shrub canopies may also protect seedlings from access by herbivores. In the intensively arid zone, seedlings likely experienced relatively large benefits from protection by the structure of established shrubs [34]. This would result in much higher seedling survival rates inside shrub canopies versus outside, and this is indeed what we observed [14]. Thus recruitment only appears possible inside canopies of established shrubs in this zone. These recruitment patterns reflect important mechanisms likely driving both the random spatial pattern of *C*. *stenophylla* in the semi-arid zone and its clumped spatial patterns in the arid and intensively arid zones.

Water availability is one of the main limiting factors for plant establishment and growth in arid environments. Water-saving strategies are key characteristics for plants growing in arid conditions. Transpiration rates of outer-canopy leaves were higher in the intensively arid zone than in the semi-arid zone, probably due to the high temperature, strong solar radiation and severe water deficit of air in the intensively arid zone. Water use efficiency (WUE) of *C*. *stenophylla* was higher in the intensively arid zone than in the semi-arid zone, which reflects a water-saving strategy of *C*. *stenophylla* in highly arid environments.

In the semi-arid zone, *C*. *stenophylla* were present as relatively small shrubs (or shrub clusters) with random spatial patterns, so *C*. *stenophylla* transpiration rates and WUE did not differ significantly between inner-canopy and outer-canopy leaves. In contrast, in the intensively arid zone, *C*. *stenophylla* occurred as relatively large shrub clusters with clumped spatial patterns, which are known to reduce interior wind speed and air temperature, and increased interior humidity [35, 36]. Thus transpiration rates of inner-canopy leaves were significantly lower than outer-canopy leaves, and WUE of inner-canopy leaves was significantly higher than outer-canopy leaves in the intensively zone. Higher transpiration rates among outer-canopy leaves versus inner-canopy leaves have been observed previously in other plant species [37]. Thus clumped spatial patterns may enable *C*. *stenophylla* to reduce water consumption and increase WUE significantly, and it likely enhances *C*. *stenophylla* survival under drought stress. Overall, aggregation of individuals within populations appears to be an effective and important ecological strategy that allows *C*. *stenophylla* to survive and reproduce in highly arid environments.

## Conclusions

We show that drought stress plays an important role in mediating changes in spatial patterns of *C*. *stenophylla* from random to clumped arrangements along the climatic drought gradient that spans the semi-arid to intensively arid zones within the Inner Mongolian Plateau. The main factors driving these changes in *C*. *stenophylla* spatial patterns are likely: (1) Seed density decreases and seed proportions inside shrub canopies increase as drought stress intensifies. (2) “Nurse effects” of *C*. *stenophylla* shrubs on conspecific seedlings become more important as drought stress increases. (3) Water-saving characteristics associated with clumped spatial patterns likely enhance survival and reproductive success of *C*. *stenophylla* in arid environments.

## Supporting Information

S1 Fig Point pattern analyses of C. stenophylla populations on the Inner Mongolian Plateau. (DOC)
