# Supplementary material for: Changes in Spatial Patterns of Caragana stenophylla along a Climatic Drought Gradient on the Inner Mongolian Plateau
Source: PLoS One. 2015 Mar 18;10(3):e0121234. doi: 10.1371/journal.pone.0121234 (PMC4364705; doi:10.1371/journal.pone.0121234)
Supplement: S1 Fig — (DOC) [file pone.0121234.s001.doc]

|  |  |  |
| --- | --- | --- |

**S1 Figure. Point pattern analyses of *C. stenophylla* populations on the Inner Mongolian Plateau.**

a, semi-arid zone; b, arid zone; c, intensively arid zone. Approximate 99% confidence envelopes (confidence intervals) were calculated using a Monte-Carlo simulation test with 99 randomizations. If the spatial pattern of shrubs within a quadrat is random, calculated *K(t)* values would be within the confidence envelope; however, if the spatial pattern is clumped or uniform, *K(t)* values would be either above the upper bound or below the lower bound of the envelope, respectively.
